# Raising awareness of sex and gender bias in artificial intelligence and health

**DOI:** 10.3389/fgwh.2023.970312

**Published:** 2023-09-06

**Authors:** Nataly Buslón, Atia Cortés, Silvina Catuara-Solarz, Davide Cirillo, Maria José Rementeria

**Affiliations:** ^1^ Life Sciences Department, Barcelona Supercomputing Center, Barcelona, Spain; ^2^ Women’s Brain Project, Guntershausen, Switzerland

**Keywords:** gender bias, AI, health, bias in science, gender policies

## Abstract

Historically, biomedical research has been led by and focused on men. The recent introduction of Artificial Intelligence (AI) in this area has further proven this practice to be discriminatory for other sexes and genders, more noticeably for women. To move towards a fair AI development, it is essential to include sex and gender diversity both in research practices and in the workplace. In this context, the Bioinfo4women (B4W) program of the Barcelona Supercomputing Center (i) promotes the participation of women scientists by improving their visibility, (ii) fosters international collaborations between institutions and programs and (iii) advances research on sex and gender bias in AI and health. In this article, we discuss methodology and results of a series of conferences, titled â€œSex and Gender Bias in Artificial Intelligence and Health, organized by B4W and La Caixa Foundation from March to June 2021 in Barcelona, Spain. The series consisted of nine hybrid events, composed of keynote sessions and seminars open to the general audience, and two working groups with invited experts from different professional backgrounds (academic fields such as biology, engineering, and sociology, as well as NGOs, journalists, lawyers, policymakers, industry). Based on this awareness-raising action, we distilled key recommendations to facilitate the inclusion of sex and gender perspective into public policies, educational programs, industry, and biomedical research, among other sectors, and help overcome sex and gender biases in AI and health.

## Introduction

Artificial intelligence (AI) is pervasive in our everyday lives, and increasingly so in the health domain, from biomedical research to clinical practice (1). Nevertheless, growing evidence of AI reflecting and perpetuating discrimination based on personal attributes, such as sex and gender (see Box 1), is leading to an increased attention to this issue (2) (see Box 1). Addressing bias in AI for biomedical applications requires consideration of ethical, societal and technical aspects that have an impact on the design, deployment and use of AI-based systems (3). Hence, achieving satisfactory outcomes in conventional metrics, such as classification accuracy, is no longer sufficient. To gain insights into health and advance Precision Medicine, it is crucial to factor in the unique biological, behavioral, and environmental characteristics of each individual, including sex and gender that can influence health outcomes and the development of diseases (3).

Box 1 **Definitions of sex and gender (CIHR Institute of Gender & Health. Available online: https://cihr-irsc.gc.ca/e/48642.html)**

**Sex** refers to a set of biological attributes in humans and animals. It is primarily associated with physical and physiological features including chromosomes, gene expression, hormone levels and function, and reproductive/sexual anatomy. Sex is usually categorised as female or male but there is variation in the biological attributes that comprise sex and how those attributes are expressed.
**Gender** refers to the socially constructed roles, behaviours, expressions and identities of girls, women, boys, men, and gender diverse people. It influences how people perceive themselves and each other, how they act and interact, and the distribution of power and resources in society. Gender identity is not confined to a binary (girl/woman, boy/man) nor is it static; it exists along a continuum and can change over time. There is considerable diversity in how individuals and groups understand, experience and express gender through the roles they take on, the expectations placed on them, relations with others and the complex ways that gender is institutionalised in society.

The entire and equal participation of women in all areas of society, along with the development of AI-based systems and their fair representation in health data, is a fundamental human right as asserted by two pivotal documents that followed the Universal Declaration of Human Rights (4). The Convention on the Elimination of all Forms of Discrimination Against Women (CEDAW) (5), adopted by the United Nations (UN) in 1979, explicitly articulates that any discrimination against women, including that perpetuated by biased AI-based systems, “violates the principles of equality of rights and respect for human dignity”. The Beijing Declaration and Platform for Action (6), adopted by the UN in 1995, presents a global policy framework and guidance for action to effectively realize gender equality.

Enhancing women's representation in science, technology, engineering, and mathematics (STEM) disciplines, especially computational biology and bioinformatics, is crucial to fight existing and emerging sex and gender bias in AI and health. Underrepresentation of women in these fields has been largely reported (7, 8) and a number of initiatives, increasingly focused on intersectionality (9, 10), have been created. Forms of discrimination against women have been reported in several aspects related to computational biomedical research, including mainstream domain conferences (11), career transitions (12), citations in high-impact journals (13), among many others. Within this framework, the Barcelona Supercomputing Center (BSC) has created Bioinfo4Women (B4W) (14), a non-profit initiative of the Life Sciences Department that began its activities in 2018. In line with the BSC gender equality plan and the United Nations (UN) Sustainable Development Goals (SDGs) (15) (Supplementary Figure S1), B4W aims to increase the representation and visibility of women in bioinformatics and computational biology, specifically in high-level professional positions, through mentorship, training, conferences, and seminars. B4W also focuses on researching and addressing sex and gender biases in the application of AI to biomedicine and healthcare.

**Figure 1:**
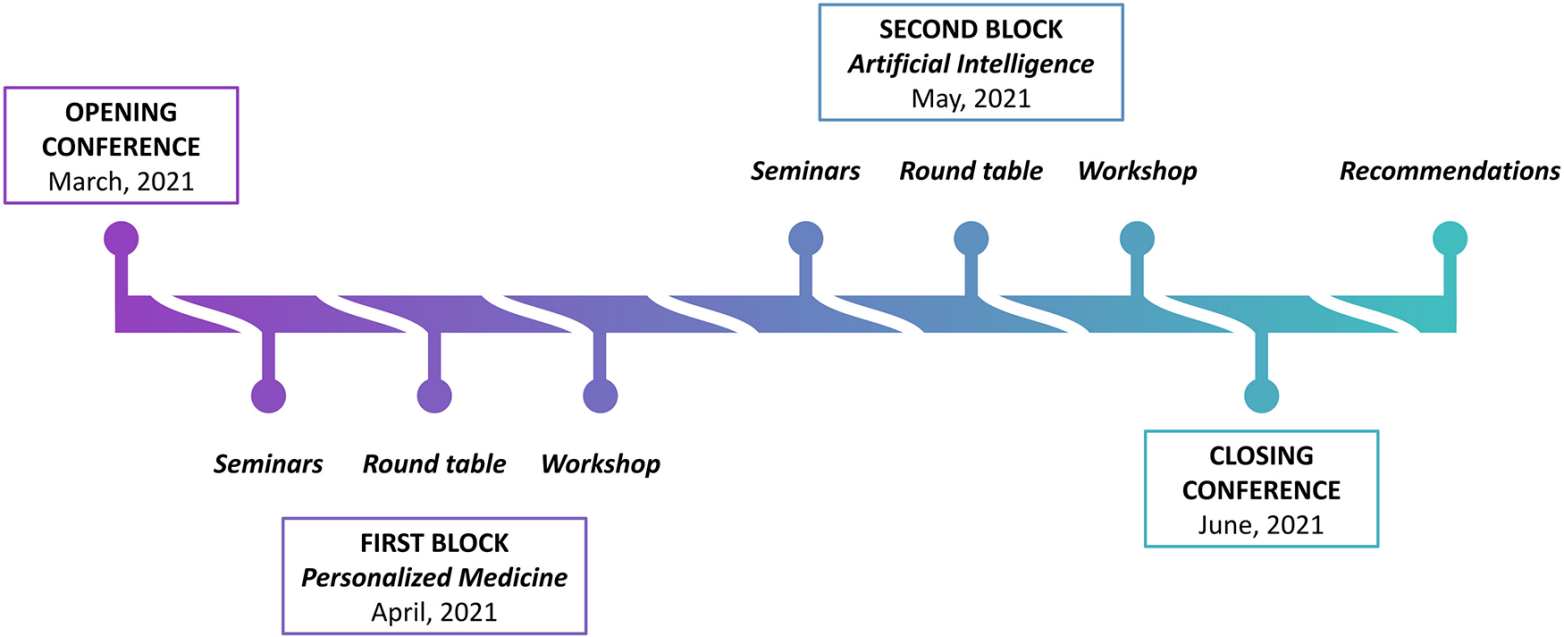
Infographic representing structure, timeline and outcomes of the B4W series of conferences “Sex and Gender Biases in Artificial Intelligence and Health” (Barcelona, March-June 2021). The series of conferences consisted of an opening conference, two blocks of activities focused on two themes (Personalized Medicine and Artificial Intelligence), and a closing conference. The main outcomes were distilled into recommendations generated after the end of the event. The detailed calendar of the series of conferences and participating domain experts are provided as Supplementary Table S1.

In 2020, the B4W designed the project “Sex and Gender Biases in Artificial Intelligence and Health”, in collaboration with La Caixa Foundation, to provide an interdisciplinary framework for reflection about challenges and social problems that impact the population. Thus, during the year 2021, the project took off as a unique series of conferences, in which international experts and citizens collaborated with the common objective of raising awareness about sex and gender bias in AI and health. The conference series included nine hybrid events, from March to June 2021 (Figure 1), consisting of keynote sessions and seminars followed by round tables open to the general public, and two workshops with international invited experts from different professional backgrounds [biomedical researchers, engineers, sociologists, representatives of Non-Governmental Organizations (NGOs) and the industry, journalists, lawyers, policy-makers].

In this work, we summarize and discuss the main outcomes of this event, especially the two workshops, and the relevance of the identified solutions to raise awareness about sex and gender bias in AI and health. Based on the acquired knowledge, we identified four major strategic areas of action (lack of data; social impact and awareness; AI biases; regulatory aspects) and proposed specific recommendations, which focus on scientific, educational, and political strategies (e.g., enhancing women's representation in STEM fields) that would help overcome sex and gender bias in AI and ultimately benefit women's health.

## Methods

### Organization of the series of conferences

The series of conferences titled *“*Sex and Gender Biases in Artificial Intelligence and Health” was developed and organized by the B4W with the support of La Caixa Foundation (16). This initiative was carried out over four months, starting on 16th March 2021, and ending on 16th June 2021 (Supplementary Table S1). The series kicked off with an opening conference where members of B4W presented the programme, agenda, and objectives, with contributed talks from public institutions as well as from recognized researchers in the area. The two following conferences, held in April and May, tackled the problem of sex and gender bias from two perspectives: personalized medicine and AI. The topics were debated in the form of seminars and round tables open to the public, thus providing a collective space for reflection. Two closed participatory workshops with invited experts on the subjects subsequently followed the previous activities on the same days. Recommendations have been generated based on the outcomes of such workshops after the end of the series in June when a final conference summarized the main conclusions that emerged from the event. A depiction of the outlined process is reported (Figure 1). All the activities, except the closed participatory workshops, were recorded and are available, together with further information about the event, at the B4W website (17).

### Design thinking workshops

The methodology applied in the two workshops of the series of conferences was based on Design Thinking (18, 19), a technique consisting of collaborative sessions that are used to innovate and focus on the needs of people. The work process has five steps that are recommended as key to the co-creation of research: empathize, define, ideate, prototype and test. The process considers both the socio-ethical and the technological aspects and ensures that the proposed innovative solutions are viable and sustainable. This methodology of co-creation and participatory research typically seeks to balance interests, benefits and responsibilities between the relevant stakeholders, focus attention on user opinions, needs, and make the whole process from planning to implementation, with a transparent and inclusive process with citizens (20). Practically, the discussion among the workshop participants was organized around key challenges (Supplementary Table S2) and facilitated by Co-emprèn (21) using the virtual whiteboard software Miró. The first workshop, titled “Building a future for equality: challenges and action strategies for personalized medicine”, gathered 30 experts. The second workshop, titled “Building a future for equality: a route map to an inclusive AI for a better health system”, gathered 28 experts. In both workshops, the invited participants, who contributed on a voluntary basis, were divided into 4 groups of 5 or 6 people. Each of these groups selected three challenges to discuss and exposed the proposed solutions to the others. All responses were collected using a system of codes (Lack of data; Social impact and awareness; AI biases; Regulatory aspects), reflecting the solutions provided by the specialists in the area. These proposed solutions were categorized by the authors into strategic areas of action (see Results, “Categorization of the proposed solutions”) and further examined to deduct general recommendations (see Results, “Recommendations”).

### Recommendations derived from the event

The scope of the discussion generated during the workshops was very ample and interdisciplinary. It included possible solutions and goals to overcome the existing biases related to personalized medicine and AI, exposing the underrepresentation of women in the STEM fields and decision-making positions. The responses of the participants encompassed several areas, such as measures to promote institutional and legal changes in public policies, and at the level of knowledge, to fix the data and to improve the tools and realize a more effective personalized medicine, specifically tailored to women and men, as well as in the design of new technologies and correction of algorithms considering the sex and gender dimension in scientific research. Given such a high level of complexity, we first identified four major strategic areas of action, based on the system of codes that we used to collect the information (Lack of data; Social impact and awareness; AI biases; Regulatory aspects), and then derived specific recommendations at different levels (AI practitioners and academies; the industry; the civil society; policy-makers and governments) (see Results, “Recommendations”).

## Results

### A large-scale awareness-raising event to gather solutions to key challenges

The series of conferences organized by B4W and La Caixa Foundation (Figure 1) was a sizable event with a substantial impact and a complex organization that was specifically tailored to fulfill two major objectives. The first objective was to provide an open space for reflection that not only involved specialists but, most importantly, brought the citizens in contact with them. Despite the limitations due to the COVID-19 pandemic, the hybrid format enabled this objective to be satisfactorily fulfilled. The specialists represented academic and scientific professionals (university professors and researchers, scientists, independent researchers, social science specialists), NGOs and civil society organizations (executive directors, project coordinators, social workers, volunteers), and public sector professionals (public officials, politicians, professionals specialized in government areas), mostly from western European countries. The second objective was to design a large-scale event with a tangible outcome, which is represented by the recommendations provided in this work. This objective was fulfilled thanks to the rigorous organization and execution of two Design Thinking workshops with international experts (see Methods) faced with intended challenges and key questions (Supplementary Table S2).

### Categorization of the proposed solutions

The activities of the series of conferences, and in particular the two workshops, offered the opportunity to bring people from different fields of expertise together. The Design Thinking methodology enhanced a dynamic debate in reduced groups followed by a general discussion that aimed to find the most relevant challenges and proposals to tackle them. These recommendations have a very broad scope from social, technical, ethical, regulatory and medical aspects, product of the interdisciplinary profile of the project that marks a complete approach and the results are applicable in the planning of AI in health with a sex and gender perspective. To ease the analysis, we categorized the main takeaways of the workshops into four major strategic areas of action: (1) Lack of data; (2) Social impact and awareness; (3) AI biases; (4) Regulatory aspects. The corresponding proposed solutions are provided in Table 1.

**Table 1:** Four major strategic areas of action identified in the workshops and the corresponding proposed solutions.

Strategic areas of action	Proposed solutions
Lack of data	*Best practices*. Entities such as the European Medicines Agency (EMA) and the European Federation of Pharmaceutical Industries and Associations (EFPIA) should include the sex and gender terms in the data collection forms, appropriately and consistently used based on standard ontologies, such as the Gender, Sex, and Sexual Orientation (GSSO) ontology (22).
	*Expansion of data collection*. Data sources integration should be promoted (e.g. integrating health data from primary care with social data from geographic regions, neighborhoods, local groupings). In agreement with the endorsement of data altruism in the EU Data Governance Act (23), voluntary campaigns could create repositories of inclusive data reflecting population variability and diversity.
	*Quality and balance of datasets.* Dataset quality and AI model explainability could be improved through relevant organizational, technical and security measures, such as designing unbiased data collection strategies, defining the conditions of data reutilization, investing into resources to balance the datasets and improve their representativeness.
	*Inclusive strategies in healthcare.* The gender dimension should be included in all the strategies and actions defined to improve global health (24). This encompasses the definition of inclusive strategies promoting adequate care for people belonging to vulnerable groups such as the LGBT+ community, with special attention to transgender people (25, 26).
	*Intersectionality.* Biomedical research needs to expand and include intersectional categories of sex, gender, sexual orientation, ethnicity, place of residence, socio-economic situation, disability, and age. The adequate representation of population segments that are generally underrepresented in clinical studies is key (27, 28).
Social impact and awareness	*Inclusion of socio-cultural aspects*. The 17 UN SDGs (29) are recognized as a critical tool for impact evaluation. It is essential to establish general norms that have a global perspective of the universal necessities and socio-cultural challenges, connecting with the population and creating literacy about AI opportunities, limitations and risks.
	*Targeted communication and education*. Promoting awareness of sex and gender biases in AI should complement training and education activities (e.g., university programs) targeting future professionals in STEM and health. As prompted by several initiatives (30), dedicated campaigns in schools are endorsed as well as the acquisition of relevant computational skills in primary and secondary education.
	*Awareness of AI bias and personalized medicine*. Outreach campaigns involving social movements and media influencers are identified as relevant activities. Activism against AI bias, exemplified by notable cases (31), should promote the dissemination of scientific evidence on sex and gender differences in human health to achieve fair AI-aided personalized medicine.
AI biases	*Multidisciplinarity and inclusive development teams*. Participatory methods involving the underserved communities at all the phases of the AI lifecycle are encouraged. It is important to integrate different points of view to avoid bias and to generate audits for citizen participation, as well as to focus on the social impact of AI and in relation to global goals (32).
	*Explainable and privacy-preserving AI models*. Explainability of AI models is broadly recognized as decisive to identify AI bias (33, 34). However, as concerns are raised regarding data anonymization and reidentification, such transparent AI models should preserve the confidentiality of personal and sensible data, thus the highest security standards should be enforced in data handling and model deployment.
	*Algorithmic auditing*. As recently highlighted by the Digital Regulation Cooperation Forum (DRCF) (35), reviewing algorithmic processing systems is imperative to control for discriminatory outcomes and negative societal impact of AI models. Beyond the health domain, connecting such auditing efforts with the UN SDGs enables them to focus on global issues requiring technical and legal interventions.
	*Increased quality and fairness of training datasets*. Training dataset should reflect the unaltered characteristics of the population distribution under study without any bias. Although arduous to accomplish (36), a comprehensive catalog of biases would allow AI developers to easily identify them.
	*Control and Correction Tools*. Despite advances in the implementation of effective metrics for AI bias assessment and mitigation, the field still holds several open questions (37). Authoring systems to control data characteristics and ensure citizens’ rights to privacy and protection from discrimination represent a promising route to overcoming AI bias propagation.
Regulatory aspects	*Dedicated public policies*. Political actors should look for industrial and academic synergies to enhance funds for projects on sex and gender bias in AI for health and impact assessment. Additionally, peer-reviewed publications, especially focusing on AI applications, should require study design and analytical procedures with a sex and gender perspective.
	*Strong legal schemes and certifications*. Having a strong normative development linked to article 9 of the EU General Data Protection Regulation (GDPR) (38) is considered highly critical, accounting for the inclusion of minorities in the regulatory process, the just assignment of liability, and AI governance regimes, such as certification tools (39).

### Recommendations

The main outcome of the event is the creation of recommendations deducted from the information gathered in the two Design Thinking workshops. Specifically, the qualitative analysis of the solutions to the key challenges proposed by the 58 participating experts (see Results, “Categorization of the proposed solutions”) allowed us to derive a list of recommendations that specifically target distinct stakeholders: AI practitioners and academies; the industry; the civil society; policy-makers and governments (Table 2).

**Table 2:** Recommendations deducted from the information gathered in the two design thinking workshops.

Recommendations for AI practitioners and academies
• Educate and train on how to introduce the sex and gender dimension in research and during career-specific subjects focusing on the social impact of AI and its applications for social good. • Define and apply metrics to evaluate AI biases, and techniques for mitigating biases in datasets and models. • Use adequate guidelines and international recommendations to standardize the data collection process. • Work and include a socially inclusive and interdisciplinary perspective to define cross-cutting solutions in health and AI that include health professionals, sociologists, psychologists, engineers and end-users to empower diverse teams with better results and solutions. • Promote publishing policies that foster sex and gender balance in research recruitment and analysis of data.
Recommendations for industry
• Promote the use of certification and regulation in all the processes • Provide more information to all the staff regarding the relevance of quality and fairness in training data sets. • Develop and apply methods to balance databases and make more emphasis on the documentation of AI processes and applicability. • Include socio-cultural aspects into AI processes by integrating different points of view in the design, development and evaluation of technological research in order to avoid bias and to generate audits for citizen participation.
Recommendations for civil society
• Participate in educational programs, open debates and other initiatives to grow a digital literacy and responsible scientific research and development culture in a plural and egalitarian way. • Require scientific evidence and transparency in AI processes from companies, academia, and policy-makers that have a social impact. • Develop initiatives that protect the most vulnerable population, especially women, in AI applications and health. • Promote and demand AI guidelines aligned with the UN SDGs as a goal to have clear benefits in society.
Recommendations for policy-makers and governments
• Define and offer a public certification and regulation as a key legal aspect in all sectors to guarantee the benefits of AI in society. • Provide an inclusive strategy in health promotion and care to benefit all citizens. • Develop public policies in trustworthy AI to apply in all sectors. • Invest in research and initiatives on AI regarding gender and diversity. • Provide more public information to citizens and training about how to introduce the perspective of sex and gender in science.

### Comparison with related work

A highly debated topic in the two workshops focused on the enhanced inclusion of women and diverse perspectives in STEM disciplines as a potential factor to break biases in AI (see Table 1, “Social impact and awareness”). A large amount of suggested actions to broaden participation in STEM can be found in the literature. Recommendations have been compiled by women in STEM (40), specialists in distinct fields [e.g., women in immunology (41)], or international agencies such as UNESCO (42). Moreover, recommendations have been systematically reviewed from national reports (43), and synthesized from conferences focusing on specific fields [e.g., women in neuroscience (44)]. Commonly, surveys are used as effective tools to distill recommendations, such as those on gender transformation in global science provided by the International Science Council (ISC) and collaborators (45). The ISC recommendations are based on the results of an extensive online survey conducted among over 250 organizations around the globe, including science academies, international disciplinary unions and associations covering engineering, medical and social sciences (45). Due to their wide scope, we used the ISC recommendations as a baseline to qualitatively assess the level of agreement and complementarity with B4W recommendations and mission (Table 3).

**Table 3:** Recommendations form a study on gender equality by the international science council (ISC) and collaborators (45) compared with B4W recommendations and mission.

ISC recommendation	Description	Comment
Extension of survey	The current recommendations should be complemented by the voices of more global science organizations.	B4W recommendations are based on the voices of experts from different scientific and technological organizations globally.
Analysis of gender-related organizational policy, structure and actions	Deeper analyses of models of policy, structure and action and the identification of best practices should be encouraged.	B4W recommendations endorse the definition of best practices and public policies to invest in research and initiatives regarding sex and gender perspective in AI.
Development of a central repository	A central repository of gender-related policies and actions should be created.	B4W supports the archiving of gender-related policies following the BSC gender equality plan.
Incorporation of regional considerations	Plans to utilize regional presence to gain insights and coordinate national actions to advance gender equality should be promoted.	B4W is mainly active at national level. Our activity is an example of local promotion of gender equality.
Advancing women to leadership positions	Women's leadership and service in governing bodies should be supported.	B4W aims to increase the representation and visibility of women in bioinformatics and computational biology, specifically in high-level professional positions.
Consideration of diversity, intersectionality and inclusivity	Actions to raise awareness about the need for diversity and inclusivity in global science, with a focus on intersectionality and gender, should be taken.	B4W recommendations promote the adequate representation of intersectional segments of the population that are generally underrepresented in health data used for AI development.
Analysis of discipline-based gender transformation	Discipline-based actions are needed to increase the number of women researchers.	B4W recommendations bring forward the inclusion of socially inclusive and interdisciplinary perspectives to define transversal solutions in health and AI.
Establishment of monitoring and evaluation frameworks	Surveys should be conducted on a regular basis to track gender transformation.	B4W recommendations consider certifications as a key legal tool to guarantee fair AI development and application.
Identification of lessons from young academies	As young academies are significantly more gender-balanced than senior academies, follow-up studies on women in leadership positions are needed.	B4W promotes the research done by women in computational biology, with special focus on their transition from postdoc to junior independent positions.
Shift from a focus on “numbers” to institutional and knowledge transformation	Gender transformation should focus on institutional culture and knowledge production embracing the sex and gender dimension in all activities.	B4W recommendations support the dedicated educational programs, open debates and other initiatives to grow a digital literacy and responsible scientific research and development culture.

## Discussion

AI systems are able to deliver cost-effective, dynamic access to health information and efficient handling of complex problems related to human diseases. However, if the application of such technologies results in unfair and unjust outcomes, they can propagate inequality and discrimination in our society, especially against women and other underserved communities, having a severe impact on the health system and the lives of women and men (46–48). The emergence of sex and gender bias in AI and health is linked to several unresolved issues, such as the diversity gap in clinical trials (49, 50), poor data management (51, 52), the lack of data collection accountability (53, 54), among many others. Thus, different strategies need to be defined to help the society and the experts understand and address these issues, especially in high-stakes AI applications (55, 56). Raising awareness and improving social impact by involving all stakeholders impacted by AI is of the highest relevance to avoid inequality and human's rights violations. In particular, the role of policy-makers and governments is fundamental to implement expert recommendations. Policies should boost research and development in sex and gender issues in health and AI following ethical values, normative environments, educational strategies, certification frameworks, and a transparent the financial flow requiring AI trustworthiness in requests and tenders of funds, as recently indicated by the Ethical Funding for Trustworthy AI framework (EFTAI) (57).

Dedicated initiatives at institutional and research level, such as B4W, represent effective ways to discuss, investigate and find solutions to sex and gender bias in AI and health. One of the main objectives of B4W is to promote biomedical research that includes the sex and gender perspective to highlight the relevance of these categories in medical research and technological development to achieve a fair realization of personalized medicine.

The conference series “Sex and Gender Bias in Artificial Intelligence and Health*”*, organized by B4W in collaboration with La Caixa Foundation, represented a unique opportunity to introduce social, ethical, legal and technical challenges of AI, providing a interdisciplinary perspective to a broad and diverse audience. Topics of gender diversity in STEM disciplines and the impact of sex and gender biases on women's health were discussed. The event ecompassed a variety of activities including keynotes, open debates and Design Thinking workshops, providing a space for open debate among different domain experts and the society on sex and gender bias in AI and health. The main outcomes of the event can be summarized in a call of action to include the sex and gender dimension in research and technology to guarantee scientific quality and excellence.

The Design Thinking methodology applied to the workshops allowed us to obtain insights from different perspectives, discussing risks and opportunities in an egalitarian environment. This project showed the relevance of defining participatory strategies regarding AI aiming to raise awareness and define a roadmap to apply discipline-specific measures and social impact assessment. Following the schemes established during the sessions, specific actions to change in the present situation were identified at different levels of science, citizenship, and the public political sector, which resulted in a series of recommendations that are reported in this work. Also, it is essential to acknowledge the inherent limitations of such an initiative and its outcomes, mainly related to challenges associated with inclusivity, limited reach, and skewed perspectives.

As the organization of the event itself demonstrated, promoting collaboration and debate among different experts and networks that could contribute to knowledge generation and to inspire and promote research projects with a social impact is crucial to raise awareness about the issue of sex and gender bias in AI and health. As a result, B4W members have been subsequently invited to participate in initiatives such as Women in Data Science (WiDS Barcelona) (58), and the Association for Computing Machinery's Council on Women in Computing (ACM-W) chapter (59). Moreover, bringing society closer to the health implications of sex and gender biases in AI helps identify and make innovative proposals to address them, inspiring and guiding future work with a more inclusive approach. Finally, implementing public policies, affirmative actions, and legislation will allow underrepresented communities, such as women and girls in STEM, to effectively participate in the workforce that will produce the new generations of AI systems (60–62). In this regard, B4W was invited to the Catalan Parliament to expose the conclusions of the event and co-authored a study report for the Panel of the Future of Science and Technology (STOA) of the European Parliament (54).

## Data Availability

The original contributions presented in the study are publicly available. This data can be found here: https://bioinfo4women.bsc.es/sex-and-gender-bias-in-artificial-intelligence-and-health/.
