# Supplementary material for: Raising awareness of sex and gender bias in artificial intelligence and health
Source: Front Glob Womens Health. 2023 Sep 6;4:970312. doi: 10.3389/fgwh.2023.970312 (PMC10512182; doi:10.3389/fgwh.2023.970312)
Supplement: Supplementary file 1 [file Datasheet1.pdf]

Raising Awareness of Sex and Gender Bias in Artificial Intelligence and Health

Nataly Buslón[1,*,†], Àtia Cortés[1,†], Silvina Catuara Solarz[2], Davide Cirillo[1], Maria José Rementeria[1,*]

[1] Barcelona Supercomputing Center, Life Sciences Department, Barcelona, Spain
[2] Women's Brain Project, Guntershausen, Switzerland

† These authors have contributed equally to this work and share first authorship

* Correspondence:
Nataly Buslón
nataly.buslon@bsc.es
María José Rementeria
maria.rementeria@bsc.es

Supplementary Figures

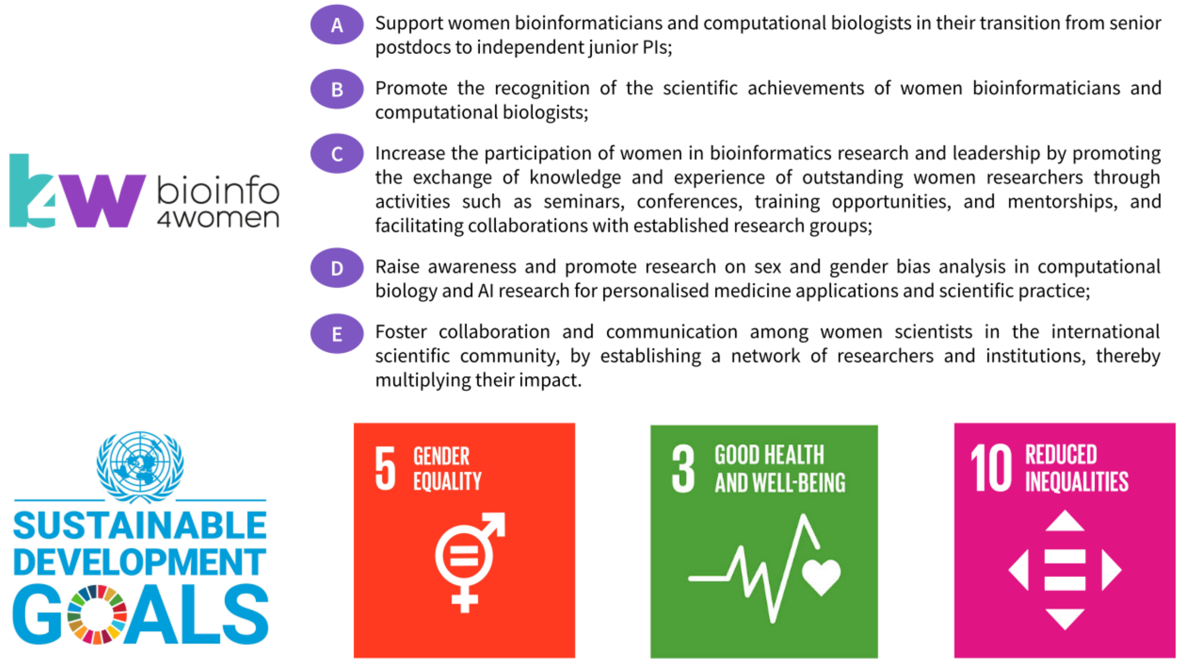

Supplementary Figure 1. The objectives of the Bioinfo4Women (B4W) program of the Barcelona Supercomputing Center in relationship to three main United Nations' Sustainable Development Goals: Goal 3 ("Health and Well-being"), Goal 5 ("Gender equality") and Goal 10 ("Reduction of inequalities")

Supplementary Tables

Supplementary Table 1. Detailed calendar of the series of conferences and participating domain experts.

Date	Description	Speakers
March 16th, 2021	Opening conference	Welcome speech:<br>Barbara Palacio, director of the Palau Macaya from La Caixa Foundation<br>María José Rementeria, principal investigator at BSC<br>Nataly Buslón, Equity, Diversity & Inclusion Officer at BSC<br>Zulema Altamirano, director of the Women and Science Unit of the Spanish Ministry of Science and Innovation<br>Laura Pérez Castaño, Deputy Mayor for Social Rights, Global Justice, Feminism and LGTBI at the Barcelona City Council<br>Keynote speakers:<br>Alfonso Valencia, CREA Research Professor, director of the Life Sciences Department of BSC and director of the Spanish National Bioinformatics Institute (INB-ISCIII)<br>Maria Teresa Ferretti, co-founder and scientific director of the Women's Brain Project
April 14th, 2021	First block: *Personalized Medicine*	Open seminar ("*The perspective of sex and gender in the future of Personalised Medicine*"):<br>Carme Valls Llbet, director of the Women, Health and Quality of Life program at the CAPS Center for Health Analysis and Programs<br>Valeria Raparelli, from the University of Alberta in Canada and professor at the University of Ferrara<br>Luis Rocha, director of the NSF-NRT Complex Networks and Systems graduate program in computing, member of the Indiana University Network Science Institute<br>Silvina Catuara Solarz, EIT Health member of the WHO roster of experts in digital health and researcher of the Women's Brain Project<br>Round table:

		moderated by Davide Cirillo, principal investigator at BSC<br><br>Closed workshop ("*Building a future for equality: challenges and action strategies for personalized medicine*"):<br><br>30 participants
May 11th, 2021	Second block: *Artificial Intelligence*	Open seminar, "*Social, Ethical and Technical challenges of sex and gender bias in AI and healthcare*":<br><br>Karina Gibert, Professor at the Universitat Politècnica de Catalunya, co-founder and director of the IDEAI-UPC group and vice president of Equity and Ethics at COEINF<br><br>Itziar de Lecuona, associate professor at the University of Barcelona School of Medicine, deputy director of the Bioethics and Law Observatory and Chair of Bioethics at UNESCO<br><br>Allison Gardner, lecturer at Keele University, representative of Women Leading in AI and member of the IEEE Standard Association<br><br>Paula Petrone, Associate Research Professor at ISGlobal and Founder and CEO at Phenobyte<br><br>Round table:<br><br>moderated by Àtia Cortés, recognized researcher at BSC<br><br>Closed workshop ("*Building a future for equality: a route map to an inclusive AI for a better health system*"):<br><br>28 participants
June 16, 2021	Final conference	Farewell speech:<br><br>María José Rementeria, principal investigator at BSC<br><br>Josep Maria Martorell, associate director of BSC<br><br>Keynote speakers:<br><br>Londa Schiebinger, John L. Hinds Professor in History of Science in the Department of History at Stanford University and director of the Gendered Innovations EU / US project in Science, Health, Medicine, Engineering and Environment<br><br>Catherine D'Ignazio, associate of Planning and Urban Science in the Department of Urban Studies and Planning at MIT and director of the Data + Feminism Lab

Supplementary Table 2. Challenges and key questions presented to the workshop participants.

**Workshop**	**Challenge**	**Key question**
"*Building a future for equality: challenges and action strategies for personalized medicine*"	Lack of data	How might we provide solutions to the lack of data to avoid sex and gender issues?
	AI biases	How can we avoid algorithm (AI) biases?
	Misuse of data	What solutions can be defined to prevent misuse of data? (manipulations, control, exclusion of minorities, ...)
	Inequality	What actions could be taken in personalized medicine and AI to avoid social inequalities (in gender, income, race, age, ...)?
	Privacy	How might we guarantee patient's data privacy in personalized medicine?
	Awareness	How might we make a given target group (citizens, biomedical researchers/physicians, companies/laboratories/technologists, governments) more awas of the importance of sex & gender biases?
	Communication	How might we best disseminate these issues to beat sex & gender biases in health?
"*Building a future for equality: a route map to an inclusive AI for a better health system*"	Regulation	What aspects should be regulated to achieve a reliable/not biased AI?
	AI biases	How can bias be avoided (either in data, algorithms, interpretation)?
	Social impact	How can we guarantee that AI in health has a positive impact aligned with SDGs (sustainable development goals)?
	Evaluation	What evaluation methods would help generate confidence in personalized medicine based on AI?
	Awareness	What practical actions are needed to promote consciousness to different stakeholders about their rights and responsibilities in the use of AI?

		(citizens, biomedical researchers/physicians, companies/laboratories/technologists, governments)
